# Integrating a Suicide Prevention Program into the Primary Health Care Network: A Field Trial Study in Iran

**DOI:** 10.1155/2015/193729

**Published:** 2015-01-08

**Authors:** Seyed Kazem Malakouti, Marzieh Nojomi, Marjan Poshtmashadi, Mitra Hakim Shooshtari, Fariba Mansouri Moghadam, Afarin Rahimi-Movaghar, Susan Afghah, Jafar Bolhari, Shahrzad Bazargan-Hejazi

**Affiliations:** ^1^Mental Health Research Center, Tehran Institute of Psychiatry, School of Behavioral Sciences and Mental Health, Iran University of Medical Sciences, Tehran, Iran; ^2^Department of Community Medicine, School of Medicine, Iran University of Medical Sciences, P.O. Box 14155-5988, Iran; ^3^Department of Clinical Psychology, University of Social Welfare and Rehabilitation Sciences, Tehran, Iran; ^4^Mental Health Bureau, Lorestan University of Medical Sciences, Lorestan, Iran; ^5^Iranian National Center for Addiction Studies (INCAS), Iranian Institute for Reduction of High-Risk Behaviors, Tehran University of Medical Sciences, Tehran, Iran; ^6^Department of Psychiatry, University of Social Welfare and Rehabilitation Sciences, Tehran, Iran; ^7^Department of Psychiatry, Charles R. Drew University of Medicine and Science, 1731 East 120th Street, Los Angeles, CA 90059, USA; ^8^David Geffen School of Medicine at UCLA, CA, USA

## Abstract

*Objective*. To describe and evaluate the feasibility of integrating a suicide prevention program with Primary Health Care services and evaluate if such system can improve screening and identification of depressive disorder, reduce number of suicide attempters, and lower rate of suicide completion. *Methodology*. This was a quasi-experimental trial in which one community was exposed to the intervention versus the control community with no such exposure. The study sites were two counties in Western Iran. The intervention protocol called for primary care and suicide prevention collaboration at different levels of care. The outcome variables were the number of suicides committed, the number of documented suicide attempts, and the number of identified depressed cases. *Results*. We identified a higher prevalence of depressive disorders in the intervention site versus the control site (*χ* ^2^ = 14.8, *P* < 0.001). We also found a reduction in the rate of suicide completion in the intervention region compared to the control, but a higher prevalence of suicide attempts in both the intervention and the control sites. *Conclusion*. Integrating a suicide prevention program with the Primary Health Care network enhanced depression and suicide surveillance capacity and subsequently reduced the number of suicides, especially in rural areas.

## 1. Introduction

Worldwide, there has been a 65% increase in the rate of suicide in the past 45 years; approximately one million people commit suicide every year [1]. Sixty percent of all cases of suicide in the world occur in Asia. Suicide is considered the fifth cause of the loss of life in Iran [2]. Although suicide rates differ in industrialized and developing countries, all consider suicide prevention a major priority in public health [3].

Iran has a suicide rate between 1.4 and 29.6 per 100,000 [4, 5] and the lifetime prevalence of suicidal thoughts, plans, and attempts is reported as 12.7, 6.2, and 3.3%, respectively [6]. According to a report from the Death Registry Office in Iran, the rate of suicide in the Western Provinces is two to five times higher than the average rate of the country. Also, of the 27 provinces, 23 report higher rates of suicide for women than men (7.6 versus 5.1 per 100,000) [7]. This is unusual in comparison to Western societies, where the suicide rates for men are generally higher. Methods such as self-immolation, which is generally fatal and prevalent among female suicide attempters in the Western provinces of Iran could account for this cross cultural discrepancy.

Current evidence suggests that about 90% of suicide victims suffer from at least one major Axis I mental disorder, with major depression being the most common [8, 9]. According to the SUPRE-MISS study carried out in eight countries, 95.7% of suicide attempters in Iran had at least one psychiatric diagnosis, with mood disorders being the most prevalent [10]. A fifteen-year systematic review of self-immolation in Iran revealed that 8 to 60% of victims had depressive disorders, 30% had anxiety disorders, and 60 to 75% presented with trait impulsivity [11].

Depression is the most prominent risk factor for suicide [1, 12]; however, over half of suicide victims and attempters do not receive psychiatric care [13]. Those who attempt suicide often benefit from primary care only after the fact [14]. Providing timely access to mental health care [15], and raising depression awareness [16] are therefore seen as crucial in preventing suicide attempts and actualization. The role of primary care and general practice physicians in screening and providing treatment or referral to at-risk individuals is also considered prominent and integral [17, 18].

## 2. The Iranian Primary Health Care Delivery System

During the last three decades, a primary health care network has been established throughout the country in an attempt to narrow the health disparities gap between rural and urban areas. Primary health care services in rural areas are now delivered in “Health Houses” (HHs). The HHs serves between 1000 and 1500 residences in two or three villages. These are staffed by two community health workers, called Behvarz in the local language. Behvarzes come from the villages they serve and receive two years of basic training in preventive health care. A rural Health Center (HC) supports 10 to 15 HHs. An HC is staffed by one or two primary care physicians, two health technicians, and, on occasion, by a nurse. The service area for each rural HC consists of 10,000 to 15,000 residents. Health Houses serve as the first point of contact with the community in the rural areas. As needed, patients are referred to rural HCs or to a district hospital.

In urban areas, this network is not well developed and for the most part functions through urban health posts and health centers, which recruit health volunteers to work with Behvarzes. The health volunteers have some level of high school education, are recruited from their own districts, and serve as the intermediaries between fifty to eighty households in their neighborhood and the health center in their district. They receive basic health care training for two months during which they learn how to fill out health information cards for the households they visit and deliver it to the health technicians.

The health volunteers are tasked with maintaining regular contact with the families, monitoring their health and communicating their health needs to the health technicians who work in the district's HC. They are also trained to provide health promotion and family planning-related health information to members of their community. While health volunteers receive no formal salary, they do receive health supplies, and along with their families, receive free or priority health treatment or other health care services [19, 20]. However, health volunteers serve only approximately 30% of urban areas. The entire primary health care network is administered and managed by District Health Centers, which are accountable to the Ministry of Health and Medical Education.

The overall goal of the current study is to describe and evaluate the feasibility of integrating a suicide prevention program with Primary Health Care (PHC) services in reducing the access gap in mental health care services. Integrated care is defined as a set of coordinated, patient-centered care delivered by a range of multiprofessional and informal care services [21, 22]. More specifically, we aimed to (1) increase the accurate identification of cases with major depression at the intervention sites compared to the control site, (2) document differences in reduced rates of suicide at the intervention site compared to the control site, and (3) report demographic factors associated with suicide attempt and suicide acts.

## 3. Methods

### 3.1. Study Design

This was a quasi-experimental trial in which the unit of assignment was the community: the community that was exposed to the intervention versus the community that was not exposed. This design is also called a community trial [23].

### 3.2. Study Sites

Study sites consist of two counties in Lorestan, a province located in Western Iran. Khorramabad serves as the intervention site and Khoohdasht as the control site. These counties were selected because of their high rate of suicide, compared to the country's average of 19 and 12 versus 6 per 100,000, respectively. Both counties have a well-established primary health care system. Even though community trial designs do not necessitate use of equivalent groups or communities for comparison, the counties in our study shared similar sociocultural backgrounds, as they were located in the same geographic area.

#### 3.2.1. The Intervention Site: Khorramabad

At the time of the study, the population of Khorramabad was 522,246, of whom 345,056 lived in cities and 176,912 in rural areas. There were 20 urban health centers and 28 rural health centers, as well as 152 Health Houses in Khorramabad. The County's primary health care services were provided by 49 General Practitioners (GPs), 180 health technicians, and 120 Behvarzes. There were also 450 health volunteers in urban and rural areas of Khorramabad. The city has one psychiatric hospital staffed by four full-time psychiatrists. There are also approximately 90 GPs in private practice.

#### 3.2.2. The Control Site: Khoohdasht

Khoohdasht province is 100 kilometers away from the intervention site, and its population at the time of the study was 211,886, of whom 99,963 lived in cities and 110,392 in rural areas. There are nine urban health centers and nine rural health centers in Khoohdasht that serve the local population. Primary health care services for the region are provided by two GPs, 14 health technicians, 134 Behvarzes, 203 health volunteers, and two full time psychiatrists.

### 3.3. The Development and Validation of a Depression Screener

We employed a checklist of 41 depressive symptoms using DSM-IV criteria from the Iranian Manual for Psychiatric Symptoms to create a screening tool with strong psychometric properties to identify depressed cases [24]. To establish content validity, the checklist was reviewed by two practicing psychiatrists and two psychologists in Khooramabad. We subsequently administered this checklist to 70 individuals living in the County. Of these, 35 were diagnosed with major depression and were recruited from a psychiatric outpatient clinic in Khooramabad. The other 35 participants had no diagnosed mental health disorders and were recruited from several outpatient specialty care clinics in Khooramabad. All participants were evaluated for depression by a psychiatrist who was blinded to our participant selection scheme.

Calculating Cronbach's alpha (0.95) tested the internal consistency of the checklist. We also calculated the Kaiser-Meyer-Olkin (KMO) measure of sampling adequacy, an index in comparing the magnitudes of the observed correlation coefficients to the magnitudes of the partial correlation coefficients. Large KMO value indicates sample adequacy for factor analysis. The KMO coefficient for our measures was 0.9, and Barttelet analysis yielded a satisfactory result (*χ* ^2^ = 1537/11, *P* < 0.001).

Using factor analysis, eleven factors emerged. The first (loneliness, hopelessness) accounted for 38% of the variance, and the remaining accounted for only 2–7% of the variances. These included worthlessness, depression/sadness, anger/irritability, vegetative signs, impatience/anxiety, sadness/not being happy, numbness/apathy, suspiciousness, feelings of guilt, and sexual problems. The correlation of 41 items with MDD diagnosis was 0.2–0.5. Eight items with a correlation equal to or more than 0.4 were selected for the final screening questionnaire. The area under the curve (AUC) of the screening questionnaire was 0.858. (*P* < 0.001). We determined an optimum cutoff score of 6.5, which had a sensitivity of 0.85, specificity of 0.70, and the positive predictive value (PPV) of 85%. The final version of the screener was administered to all the rural residents and 30% of the urban residents in the intervention site.

### 3.4. Project Related Activities and Training

We attempted to appropriately address and adequately harmonize project activities, goals, and objectives with the culture of the local population by establishing a steering committee from members of the Lorestan University of Medical Sciences (LUMS), Department of Health and Treatment, and the Department of Mental and Medical Health. The Steering Committee was charged with approving the final intervention plan and the training manual. We also revised existing depression training manuals to enhance their cultural sensitivity.

Our training of 49 GPs, 180 health technicians, and 120 Behvarzes at the intervention site was based on Waterfall model, using an approach in which work development is seen as flowing progressively downwards, like a waterfall, in several phases. In our study, the Behvarzes were trained by health technicians, and the last group by the GPs. This approach is a well-established training model in primary health care settings. We provided similar training for 50 GPs in private practice in the study area. All received a training binder consisting of a short training manual, the pharmacotherapy protocol for treatment and management of depressed patients, and depression and suicide prevention education brochures. We also held training sessions for 34 nursing staffs in the Emergency, Internal, and Surgery wards and provided them with depression and suicide prevention education brochures. In all cases, health care staff providers in the intervention region received training.

Training also focused on the proper management of symptoms and the referral of suicidal patients and their families to the Suicide Prevention Consultation Office (SPCO). The initial training took place one month prior to the launch of the depression-screening project, and was followed by a three-month booster training session. Pre- and posttest data helped to assess the quality of training.

**Figure 1:**
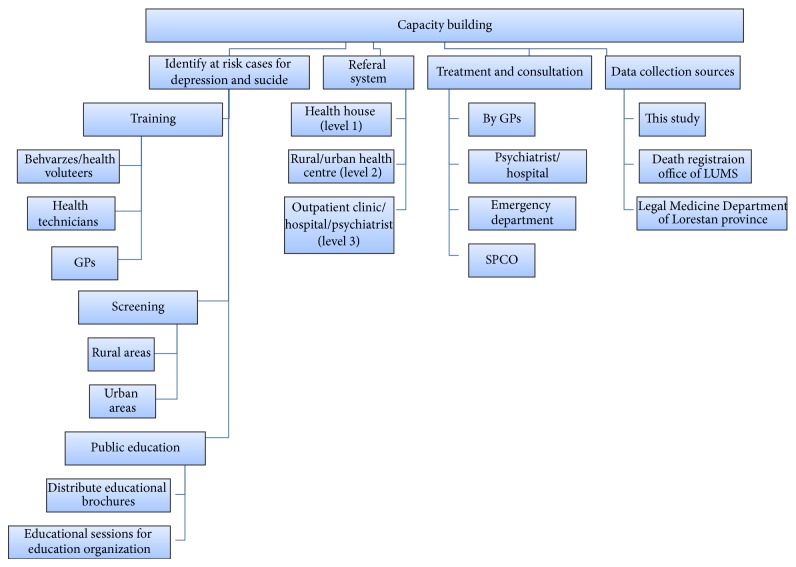
Capacity building diagram in intervention region (Khorramabad).

**Figure 2:**
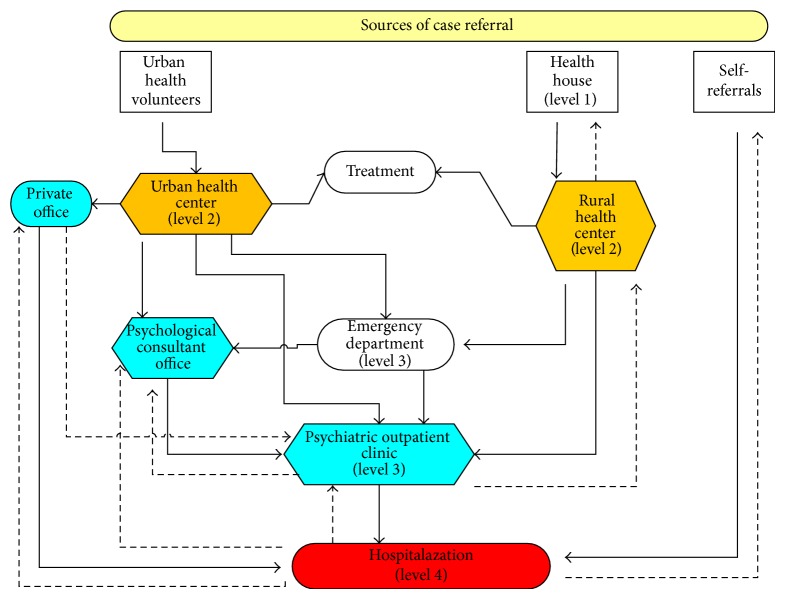
Referral pathways of individual with suicide behaviors. Three pathways of referrals were established to refer patients to final point of care: GPs/emergency department/psychiatric/outpatient clinic/hospitalization.

We also organized and established a referral pathway between different levels of health care delivery at the intervention site. These levels include Health Houses (level 1), Health Centers (level 2), psychiatric outpatient clinics and emergency departments (level 3), and the psychiatric hospital (level 4). We standardized the study data abstraction and data collection forms for each level of care (Figure 2). We held public campaigns to increase depression and suicide awareness in the local community and distributed depression-related education brochures, pamphlets, and posters at rural and urban health centers and at private practice locations. We held art exhibitions showcasing depression and suicide prevention artwork. We established a Suicide Prevention Consultation Office (SPCO) to provide consultation and referral for depressed and suicidal individuals (Figure 1).

### 3.5. The Intervention Protocol

The implementation of the intervention phase took one year (July 2006–June 2007). This followed the capacity building activities that took place between October 2005 and May 2006. The latter included creating the screening questionnaire, establishing the LUMS steering committee, training the health staff, organizing the referral pathways and data collection sites, and establishing the “Suicide Prevention and Consultation Office” (SPCO).

At each level of care, the following activities took place at the intervention site.

At the Level I care in the HHs, Behvarzes (1) screened the population in their regions, (2) completed patient-related data forms, (3) provided referrals for all positive cases to receive care either from GPs in the rural HHs or at the local emergency room, and (4) provided appropriate follow-up appointments for patients to ensure proper care. Health volunteers were in charge of similar tasks and activities at the urban areas.

At Level II care in the rural health centers, health technicians processed patients who were referred from HHs. GPs in rural health centers subsequently provided appropriate physical and mental care to these patients and referred complex cases or suicidal individuals to the outpatient psychiatric clinic in the urban area. Behvarzes then made appropriate treatment follow-up to ensure that patients sought and obtained the recommended care.

At Level III care in the emergency room, the in-charge nurse and GP were responsible for referring those who attempted suicide to the Suicide Prevention Consultation Office (SPCO) after stabilizing their medical condition. When the physical condition of the suicide attempters did not allow them to leave the emergency room, the SPCO psychologist initiated mental care treatment there.

At Level IV care in the SPCO, the licensed psychologist was responsible for providing consultation services to the depressed or to suicide attempters and their families. The first five consultation sessions were offered to patients free of charge. The SPCO psychologist made appropriate follow-up telephone calls with all patients. Patients and their families also received depression and suicide-related education brochures.

#### 3.5.1. The Standard Care Protocol

The treatment of any case of depressive disorder and suicide attempt in the Khoohdasht region took place in the established network of PHCs, as did the provisioning of psychiatric care as usual care.

### 3.6. Study Outcome Variables

The first outcome variable was the number of those who committed suicide. This information is collected at Health Houses and by GPs who come in contact with such cases. These are then reported to the County Office of Mental Health. There, duplicate data is omitted and the results are transferred to the Mental Health Bureau (MHB) at the Lorestan University of Medical Sciences (LUMS) for final evaluation. We also obtained this information from the Death Registry Office (DRO) at LUMS and the Department of Legal Medicine, which is regulated by the Lorestan judiciary system. The second and third outcome variables were the number of documented suicidal attempters and the number of identified depressed cases, both collected via active screening by the study Behvarzes in rural areas and health volunteers in the urban areas.

### 3.7. Statistical Analysis

As this was a community trial design with similar demographic characteristics across the communities, we first compared differences in the outcome variable (i.e., rate of suicide attempts) before and after the intervention within each community and then compared the differences across the two communities. The data was analyzed using SPSS for Windows, version 11.5 (SPSS Inc., Chicago, IL).

## 4. Results

### 4.1. Prevalence of Committed Suicide in the Intervention and Control Regions

During the interventions, 73 cases of suicides were reported in our study regions of which 33 were committed in Khorramabad (the intervention site) and 40 in Khoohdasht (the control site) (Table 1). After one year of intervention, the rate of suicide completion was 6.3 per 100,000 in Khorramabad (the intervention region) and 18.9 per 100,000 in Khoohdasht (the control region) (Table 2) (*P* < 0.005). The rate of suicide completion in the intervention region was reduced from 12.5 persons per 100,000 in 2007 to 6.3 in 2008, close to the average rate in the entire country. The control region, however, experienced smaller reductions, from 19.3 persons per 100,000 in 2007 to 18.9 in 2008 (*P* < 0.05).

Demographic characteristics of the suicide cases are reported in Table 1. In general, suicide was more prevalent in (1) women, (2) among those 15–25 years old, (3) in singles, and (4) among housewives, at both sites. Suicide was more prevalent in rural than urban areas both in Khorramabad (26 versus 7 cases) and Khoohdasht (22 versus 18 cases).

Higher percentages of suicide occurred during fall and winter. Of those who committed suicide, 12.6% had a history of suicide attempts, and 36.6% had a chronic mental and/or physical disorder. The most common method of committing suicide was self-immolation (42% in Khorramabad and 51% in Khoohdasht), followed by gunshot (15% and 23%), and hanging (20% and 21%) (results not shown).

### 4.2. Prevalence of Suicide Attempts in the Intervention and Control Regions

During the study period, 1293 subjects attempted suicide in both cities (Khorramabad = 1060, Khoohdasht = 233) (Table 3 and Table 4). This translated into 203 cases in the intervention site and 110 in the control site per 100,000. (*χ* ^2^ = 58.4, *P* < 0.5). The intervention/control ratios of suicide attempt were 0.1 : 0.006%. Suicide attempt was most prevalent in the 15–24 year-old age group (60%), and in urban, rather than rural areas in both Khorramabad (812 versus 244) and Khoohdasht (159 versus 74). Suicide attempts were also more prevalent in summer, autumn, and winter. The most common means of suicide attempt in both Khorramabad and Khoohdasht were drug poisoning (71 and 72%), followed by phosphorus poisoning (3 and 7%) and self-immolation (6 and 12%). (Results not shown).

### 4.3. The Prevalence of Major Depressive Disorder in the Intervention and Control Regions

Based on the field notes, approximately 60% of rural catchment areas were screened for MDD and nearly 50% of screened individuals completed scheduled, follow-up appointments. Thirty percent (30%) of the urban catchment areas were covered by health workers in cities.

During the intervention, 538 subjects with depressive disorders were identified; 525 (97.6%) patients came from the intervention site and 13 (2.4%) from the control site (*χ* ^2^ = 14.8, *P* < 0.001). The rural urban ratios of depressive disorder in the intervention site were 0.18 : 0.06 percent (*χ* ^2^ = 29.5, *P* < 0.0001).

## 5. Discussion

Our results show that increasing capacity for PHCs to actively engage in the screening and identification of at risk individuals through integrated care was not fully feasible and effective. With respect to capacity training and engagement of health personnel in the intervention, our integrated model was successful. However, our model was less effective at level I care, which involved screening the whole population for depression by trained Behvarzes. This was due mainly to time constraints and Behvarzes' heavy workload.

We found a reduction in the rate of suicide completion in the intervention region compared to the control site. But, we detected a higher prevalence of suicide attempts in both the intervention and the control sites (32 and 5.8 times more, resp.). This suggests that our capacity training increased competency of health personnel in identifying and managing higher number of individuals at risk for depression and suicide. Post-intervention comparison between the intervention and control sites revealed that 203 versus 110 suicide attempter, per 100'000 population, were identified and received needed mental care services, respectively.

Similarly, in Khorramabad, we found a higher prevalence of depressive disorders in urban (0.06%) and rural (0.18%) areas, compared to the control site. The higher number of suicide attempts and depressive disorders at the intervention site could be the result of improving and enhancing the ability of these sites to identify and capture such data, as opposed to existing capabilities at the control site.

The screening rate for depressive disorders in our study was similar to those previously reported in Iran (between 0.07 and 2%) [25]. Considering that the point prevalence of severe MDD is 2.5% percent [26, 27], our findings reveal that the screening outcome of Behvarzes was limited. In the rural area, the screening rate for MDD was 0.18%, which is far less than the real prevalence of disease. It is possible that stigma attached to mental disorders prevented individuals from reporting their symptoms, therefore limiting the ability of the Behvarzes to identify MDD cases, particularly if the Behvarz was a the local dweller. Another possible explanation for low screening outcomes could be the high, routine workload of Behvarzes. This might make it impossible to screen entire rural areas for MDD during the intervention period. Based on the field notes, approximately 60% of the intervention region was covered by the end of project; nevertheless, the rate of screening coverage was larger in the rural regions, compared to urban regions.

Given the above limitations, our intervention made it possible for the Behvarzes to refer and follow up with identified cases of MDD and suicide attempter, so that the SPCO personnel could make early contact with them in the emergency room and to provide needed mental health services. Offering immediate mental counseling to these patients often helps to prevent reattempts [28, 29].

Despite the aforementioned limitations, our results add to existing evidence. Integrating mental health care with existing primary care improves access to mental health services, expands detection and identification of patients at risk for mental disorders, and provides better management for patients with diagnoses of mental disorders [30, 31]. Our findings also suggest that the proper training and monitoring of informal, health care volunteers can facilitate the stepped-care structure [32] by creating a smooth linkage between patients with mental health symptoms and their primary care providers.

Many programs have attempted to improve the knowledge and skills of general practitioners (GPs) in regard to the screening, detection, and management of depression [17], and some approaches and methods appear more effective than others [33]. For example, investigators in a Swedish suicide prevention study were able to improve GP knowledge in the diagnosis and management of depression, subsequently decreasing suicide mortality rates [34, 35]. Similarly, improved depression management reduced the suicide rates of elderly residents in a Japanese rural community [36]. Anti-depressants were found to reduce suicide rates in Hungary [37] and also nonfatal suicidal attempts in a German study [38]. However, another study conducted in the United Kingdom reported that patient education combined with systematic monitoring and follow-up by the GP resulted in better depression recovery rates.

## 6. Conclusion and Future Direction

Our findings indicate that integrating a suicide prevention program with the PHC network enhanced the functionality of existing PHCs by increasing the depression and suicide surveillance capacity of the PHC and in reducing the number of suicides, especially in rural areas. Findings also suggest that improving PHC network capacity to screen, identify, and treat depressed patients is partially feasible. Complete screening of regions for MDD and at risk individual for suicide may require more resources including manpower. This needs further investigation.

We were also able to demonstrate that investing in an integrated approach that engages academicians, providers, and healthcare systems can improve the quality of the program and facilitates its implementation.

Further studies are needed to demonstrate the efficacy, efficiency, and sustainability of integrated approach in increasing mental health services for vulnerable populations, especially where access to primary care providers is limited [39, 40]. In addition, future studies with multiple control groups that measure and assess relevant variables at baseline are needed to validate our results. Future investigators should also attempt to collect comprehensive, intermediate patient, provider, and system-related data along the referral pathway. Data of interest may include patients' thoughts and ideas about suicide, as well as the number and details of any suicide attempts. Providers' data should also include attitudes, perceptions, and measured effectiveness in working as a team and in collaboration with other mental health care professionals.

System-based data collection should include primary and mental health care clinic scheduling, admitting, referrals, and other information. These data will result in more reliable statistics regarding the efficacy and efficiency of implementing our approach in integrating suicide prevention programs with the PHC network. Therefore, the screening of regions for MDD and at risk individual for suicide is not feasible unless another solution to come across.

## Figures and Tables

**Table 1:** Demographic features of committed suicide subjects (*n* = 73) in both cities.

Variables	Khorramabad		Khoohdasht	
	Number	%	Number	%
Gender				
Male	9	27.3	15	37.5
Female	24	72.7	25	62.5
Age				
<14	0	0	1	2.5
15–19	11	33.3	6	15
20–24	10	30.3	18	45
25–29	4	12.1	8	20
30–34	0	0	0	0
35–39	2	0	0	3
40–44	1	3	2	5
45+	5	15.2	5	12.5
Years of education				
Illiterate and <6	13	39.4	13	32.5
7–12	7	21.2	16	40
>12	13	39.4	11	27.5
Marital status				
Single	18	54.5	21	52.5
Married	14	42.4	17	42.5
Unknown	1	3	2	5
Job status				
Unemployed	8	24.2	6	15
Housewife	13	39.4	14	35
Self-employed	—	—	2	5
Student	4	12.1	5	12.5
Others	1	3.8	3	7.5
Unknown	7	21.2	10	25
Area of inhabitant				
Urban	7	21.2	18	45
Rural	26	78.8	22	55

**Table 2:** The frequency and ratio of suicide behaviors per 100,000 of population.

City	Frequency	Attempted suicide	Ratio/per 100,000 population	Committed suicide	Ratio/per 100,000 population
Khorramabad	Number	1060	203	33	6.3
	%	96.7		2.3	

Khoohdasht	Number	233	110	40	18.9
	%	85.3		14.7	

**Table 3:** The logistic regression analysis to evaluate the risk factors for committing suicide.

	Model 1 OR (CI)	Model 2 OR (CI)	Model 3 OR (CI)	Model 4 OR (CI)	Model 5 OR (CI)
Gender (female versus male)	1.9 (1.17–3.19)	1.6 (0.9–2.7)	1.5 (0.8–2.5)	1.6 (0.9–2.9)	1.03 (0.5–2.2)
Literacy (low versus high level)		3.3 (1.9–5.8)	3.3 (1.9–5.9)	3.2 (1.8–5.8)	2.7 (1.26–5.66)
Marital status (single versus married)			0.7 (0.4–1.3)	0.9 (0.5–1.7)	0.8 (0.34–2.02)
Age (lower versus higher)				1.01 (0.98–1.04)	0.99 (0.96–1.03)
Place of living (rural versus urban)					71.6 (34.5–148.7)

**Table 4:** Demographic features of attempted suicide (*n* = 1293) in both cities.

Variables	Khorramabad		Koohdasht	
	Number	%	Number	%
Gender				
Male	521	49.2	108	46.4
Female	539	50.8	125	53.6
Age				
<14	37	3.5	6	2.6
15–19	306	28.9	73	31.3
20–24	325	30.7	72	30.9
25–29	162	15.3	47	20.2
30–34	73	6.9	13	5.6
35–39	43	4.1	7	3
40–44	17	1.6	2	0.9
45+	97	9.2	13	5.6
Years of education				
Illiterate and <6	80	7.5	9	3.9
7–12	569	53.7	22	9.4
>12	411	38.8	202	86.7
Marital status				
Single	748	70.6	138	59.2
Married	309	29.2	94	40.3
Divorced/widowed	3	0.3	1	0.4
Job status				
Unemployed	158	14.9	26	11.2
Housewife	186	17.5	69	29.6
Self-employed	85	8	14	6
Student	148	14	24	10.3
Others	45	4.3	3	1.3
Unknown	438	41.3	97	41.6
Area of residency				
Urban	812	76.6	159	68.2
Rural	244	23	74	31.8
Others	4	0.4		
